# Cost-effectiveness Analysis of Genotype-Specific Surveillance and Preventive Strategies for Gynecologic Cancers Among Women With Lynch Syndrome

**DOI:** 10.1001/jamanetworkopen.2021.23616

**Published:** 2021-09-09

**Authors:** Jason D. Wright, Elisabeth R. Silver, Sarah Xinhui Tan, Chin Hur, Fay Kastrinos

**Affiliations:** 1 Herbert Irving Comprehensive Cancer Center, Columbia University Irving Medical Center, New York, New York; 2 Division of Gynecologic Oncology, Department of Obstetrics and Gynecology, Columbia University Vagelos College of Physicians and Surgeons, New York, New York; 3 NewYork-Presbyterian Hospital, New York, New York; 4 Division of General Medicine, Department of Medicine, Columbia University Irving Medical Center, New York, New York; 5 Division of Digestive and Liver Diseases, Department of Medicine, Columbia University Vagelos College of Physicians and Surgeons, New York, New York

## Abstract

**Importance:**

With the expansion of multigene testing for cancer susceptibility, Lynch syndrome (LS) has become more readily identified among women. The condition is caused by germline pathogenic variants in DNA mismatch repair genes (ie, *MLH1, MSH2, MSH6*, and *PMS2*) and is associated with high but variable risks of endometrial and ovarian cancers based on genotype. However, current guidelines on preventive strategies are not specific to genotypes.

**Objective:**

To assess the cost-effectiveness of genotype-specific surveillance and preventive strategies for LS-associated gynecologic cancers, including a novel, risk-reducing surgical approach associated with decreased early surgically induced menopause.

**Design, Setting, and Participants:**

This economic evaluation developed a cohort-level Markov simulation model of the natural history of LS-associated gynecologic cancer for each gene, among women from ages 25 to 75 years or until death from a health care perspective. Age was varied at hysterectomy and bilateral salpingo-oophorectomy (hyst-BSO) and at surveillance initiation, and a 2-stage surgical approach (ie, hysterectomy and salpingectomy at age 40 years and delayed oophorectomy at age 50 years [hyst-BS]) was included. Extensive 1-way and probabilistic sensitivity analyses were performed.

**Interventions:**

Hyst-BSO at ages 35 years, 40 years, or 50 years with or without annual surveillance beginning at age 30 years or 35 years or hyst-BS at age 40 years with oophorectomy delayed until age 50 years.

**Main Outcomes and Measures:**

Incremental cost-effectiveness ratio (ICER) between management strategies within an efficiency frontier.

**Results:**

For women with *MLH1* and *MSH6* variants*,* the optimal strategy was the 2-stage approach, with respective ICERs of $33 269 and $20 008 compared with hyst-BSO at age 40 years. Despite being cost-effective, the 2-stage approach was associated with increased cancer incidence and mortality compared with hyst-BSO at age 40 years for individuals with *MLH1* variants (incidence: 7.76% vs 3.84%; mortality: 5.74% vs 2.55%) and those with *MSH6* variants (incidence: 7.24% vs 4.52%; mortality: 5.22% vs 2.97%). Hyst-BSO at age 40 years was optimal for individuals with *MSH2* variants*,* with an ICER of $5180 compared with hyst-BSO at age 35 years, and was associated with 4.42% cancer incidence and 2.97% cancer mortality. For individuals with *PMS2* variants, hyst-BSO at age 50 years was optimal and all other strategies were dominated; hyst-BSO at age 50 years was associated with an estimated cancer incidence of 0.68% and cancer mortality of 0.29%.

**Conclusions and Relevance:**

These findings suggest that gene-specific preventive strategies for gynecologic cancers in LS may be warranted and support hyst-BSO at age 40 years for individuals with *MSH2* variants. For individuals with *MLH1* and *MSH6* variants, these findings suggest that a novel 2-stage surgical approach with delayed oophorectomy may be an alternative to hyst-BSO at age 40 years to avoid early menopause, and for individuals with *PMS2* variants, the findings suggest that hyst-BSO may be delayed until age 50 years.

## Key Points

**Question:**

What are the most cost-effective gene-specific screening and preventive strategies for reducing gynecologic cancer risk for women with Lynch syndrome (LS)?

**Findings:**

This cost-effectiveness economic evaluation found that optimal screening strategies varied by genotype. A novel 2-stage approach with hysterectomy and bilateral salpingectomy at age 40 years and delayed oophorectomy at age 50 years was effective and cost-effective for individuals with *MLH1* and *MSH6 *genetic variants, while hysterectomy with bilateral salpingo-oophorectomy was optimal at age 40 years for individuals with *MSH2* genetic variants and at age 50 years for individuals with *PMS2* variants.

**Meaning:**

These findings suggest that surgical decision-making should consider LS genotype for gynecologic cancer prevention and that a novel 2-stage approach may be associated with decreased cancer risk while avoiding early menopause in individuals with select genetic variants.

## Teaser

This economic evaluation analyzes the cost-effectiveness of screening and prevention strategies by genotype among women with Lynch syndrome.

## Introduction

The increased uptake of multigene panel testing for cancer susceptibility, particularly among women,^1^ has led to the identification of individuals with highly and moderately penetrant pathogenic gene variants. Lynch syndrome (LS) is a common autosomal dominant cancer syndrome occurring among 1 in 300 individuals.^2^ It has become more readily identified among individuals without cancer as genetic testing has increased based on family cancer history, including multiple LS-associated malignant neoplasms, such as colorectal, endometrial, and ovarian cancers. The estimated lifetime risks associated with endometrial cancer (EC) and ovarian cancer (OC) are as high as 48.9% and 17.4%, respectively, and EC is often a sentinel cancer that identifies new LS families.^3,4^ While pathogenic variants in the mismatch repair (MMR) genes *MLH1, MSH2, MSH6,* and* PMS2* and deletions in the *EPCAM* gene, which epigenetically silences *MSH2*, cause LS, recent data support variable cancer risks based on the altered MMR gene.^3,4^

Current recommendations for the management of gynecologic cancer for individuals with LS are nonspecific.^5,6^ The National Comprehensive Cancer Network suggests that the recommendation of EC surveillance with endometrial sampling be considered, but there is insufficient evidence for or against OC surveillance.^7^ Risk-reducing surgical treatment (RRS) involving total hysterectomy with bilateral salpingo-oophorectomy (hyst-BSO) is recommended as an option for individuals with LS after the completion of childbearing.^5,8^ However, the optimal timing of hyst-BSO is uncertain given the association of surgical menopause with decreased quality of life.

Despite heterogeneity in cancer risk by MMR gene mutation, current recommendations for LS gynecologic cancer prevention are not specific to genotype.^3,4,9,10,11^ Consequently, for patients with less aggressive phenotypes, such as individuals with *PMS2* variants, current gene-agnostic management strategies may be associated with more harm than benefit. Prior computer-based simulation modeling studies evaluating preventive strategies for LS-associated gynecologic malignant neoplasms^12,13,14^ have not accounted for gene-specific variation in cancer risks.

The aim of our study was to determine optimal (ie, effective and cost-effective) gynecologic risk-reduction strategies for the 4 MMR genes associated with LS. We developed a computational simulation model of the natural history of OC and EC for each LS genotype to evaluate several currently used risk-reducing strategies. We also introduce a novel 2-stage surgical approach involving hysterectomy with bilateral salpingectomy (hyst-BS) and delayed oophorectomy, a strategy that has been explored for women with *BRCA*-associated hereditary breast and ovarian cancer syndrome.^15^

## Methods

This economic evaluation followed the Consolidated Health Economic Evaluation Reporting Standards (CHEERS) reporting guideline for economic evaluations of health interventions and represents a cost-effectiveness simulation model. There was no patient contact. The institutional review board at Columbia University Medical Center considered this research exempt from review because the research did not meet criteria for human subjects research as defined by the 2018 Common Rule 45 CFR. §46.102. Informed consent was not required given that all data were obtained from published literature.

### Model Overview and Target Population

We developed a Markov state-transition cohort-level model of the natural history of LS-associated EC and OC using the Python programming language version 3.7 (Python Software Foundation); code is available for review.^16^ In the natural history group (ie, no intervention), the population consisted of women beginning in a healthy state and progressing through health states (including EC, OC, all-cause death, and cancer-specific death) starting at age 25 years and ending at age 75 years or death (eFigure 1 in the Supplement). The natural history model was the backbone upon which we overlaid surveillance and RRS.

In the intervention groups, the population consisted of women beginning in a healthy state at age 25 years and following the natural history model until reaching the age specified for each intervention. Current management relies on surveillance prior to RRS and can involve transvaginal ultrasound (TVUS), pelvic examination, endometrial sampling, and serum cancer antigen 125 (CA-125) testing at clinicians’ and patients’ discretion.^5,7^ Patients may continue surveillance regimens before RRS, which involves removal of the uterus, fallopian tubes, and ovaries (ie, hyst-BSO), resulting in surgical menopause if performed prior to menopause.

We examined alternate strategies by varying type and timing of RRS and age of surveillance initiation. In addition to variable ages for hyst-BSO and surveillance, we examined a novel 2-stage surgical approach: hyst-BS at age 40 years with oophorectomy delayed until age 50 years. We evaluated this strategy because preserving ovarian function until natural menopause provides quality of life benefits, although the tradeoff between these benefits and the potential risk of OC are unknown.^15,17,18,19^ Because this strategy has not yet been evaluated among individuals with LS, we conservatively assumed no decrease in OC risk associated with hyst-BS in the base case. However, salpingectomy is likely associated with a decrease in OC risk, with larger decreases for serous carcinomas. While endometrioid tumors are the most common histologic subtype of LS-associated OC, high-grade serous carcinomas account for 15% to 20% of these cancers.^20^ In sensitivity analyses, we evaluated a range of OC risk-reduction estimates (ie, 25% to 0% risk reduction) based on the prevalence of histologic subtypes thought to arise from the fallopian tubes among individuals with LS.

We assessed gene-specific outcomes for each strategy for women with a confirmed pathogenic mutation in 1 of 4 MMR genes (*MLH1, MSH2, MSH6,* or *PMS2*) (Table 1^7^). The modeled group began at age 25 years (allowing at least a 5-year run-in period) and cycled annually until age 75 years. Per current guidelines, we assumed no intervention (ie, surveillance or RRS) would be performed before age 30 years. All model inputs were derived from systematic reviews of the literature.

**Table 1.** Surveillance and Surgical Prophylaxis Strategies for Women With Lynch Syndrome^a^

Age, y			Intervention and surveillance strategy recommended at this age^7^
Hysterectomy and bilateral salpingectomy	Oophorectomy	Surveillance start	
35	35	30, Never	Yes
40	40	30, 35, Never	Yes
50	50	30, 35, Never	Yes
Never	Never	30, 35	Yes
40	50	Never	No
Never	Never	Never	No (except *PMS2*)

^a^ Surveillance was performed annually until a positive result or risk-reducing surgical treatment.

### Probabilities: Cancer Progression

Gene-specific and age-specific probabilities of developing EC or OC were derived from published literature (Table 2; eTable 1 in the Supplement).^3,9,13,14,20,21,22,23,24,25,26,27,28,29,30,31,32,33,34,35,36,37,38,39,40,41^ Until age 75 years, EC risks range from 11.8% to 48.9% and OC risks from 3.0% to 17.4%.^3^ We assumed that EC and OC diagnoses were independent events and that after 1 cancer diagnosis was made, a patient was no longer at risk of developing the other cancer. In the group with no intervention, patients diagnosed with cancer remained in a stage-specific and site-specific cancer state until death. Annual probabilities of OC and EC development were calibrated by visually comparing modeled and published incidence rates. We converted age-specific cumulative incidence rates to annual probabilities of cancer development as a starting point and gradually adjusted annual probabilities until they demonstrated adequate fit to published estimates at a given age.

**Table 2.** Parameter Estimates for Model Inputs

Parameter	%		Distribution for sensitivity analysis	Source
	Base case	Range for sensitivity analyses		
Probability				
All-cause mortality	CDC life tables (women)	NA	NA	Arias et al, 2016^21^
Surgical mortality	0.17	0.14 to 0.20	Beta	Wright et al, 2013^22^
OC surveillance (TVUS, CA-125)				
Sensitivity	60.0	54.0 to 66.0	Beta	Partridge et al, 2013^23^
Specificity	96.2	86.6 to 98.0		
EC surveillance (pelvic exam and biopsies)				
Sensitivity	91.0	81.9 to 98.0	Beta	Kwon et al, 2008^13^
Specificity	98.0	96.0 to 98.0		Kwon et al, 2008^13^
Surgical complication	3.0	1.0 to 4.4		Bhattacharya et al, 2011^24^; Roberts et al, 2011^25^
Risk ratio for OC development after salpingectomy	1.0	0.75 to 1.0	Beta	Ryan et al, 2017^20^; Cibula et al, 2011^26^; Gaitskell et al, 2016^27^; Reid et al, 2017^28^; Yoon et al, 2016^29^; Ely et al, 2017^30^
Cumulative risk of EC by age 75 y				
* MLH1*	37.0	30.1 to 46.5	Normal	Dominguez-Valentin et al, 2020^3^
* MSH2*	48.9	40.2 to 60.7		Dominguez-Valentin et al, 2020^3^
* MSH6*	41.1	28.6 to 61.5		Dominguez-Valentin et al, 2020^3^
* PMS2*	11.8	3.6 to 20.0		ten Broeke et al, 2015^9^
Cumulative risk of OC by age 75 y				
* MLH1*	11.0	7.4 to 19.7	Normal	Dominguez-Valentin et al, 2020^3^
* MSH2*	17.4	11.8 to 31.2		
* MSH6*	10.8	3.7 to 33.9		
* PMS2*	3.0	0.6 to 47.4		
EC stage distribution: no intervention				
Local	73.0	58.0 to 86.0	Normal	Howlader et al, 2019^31^
Regional	19.0	12.0 to 36.0		
Distant	8.0	2.0 to 8.0		
EC stage distribution: RRS or surveillance				
Local	86.0	72.0 to 98.0	Normal	Kwon et al, 2008^13^; Renkonen-Sinisalo et al, 2007^32^
Regional	13.0	2.0 to 25.0		
Distant	1.0	0.2 to 3.0		
EC 5-y relative survival				
Local	95.0	76.0 to 98.0	Beta	Howlader et al, 2019^31^
Regional	69.9	55.2 to 98.0		
Distant	16.8	13.4 to 20.2		
OC stage distribution: no intervention				
Local	16.0	8.0 to 20.0	Normal	Howlader et al, 2019^33^; Seiffert et al, 1993^34^
Regional	23.0	18.0 to 32.0		
Distant	61.0	48.0 to 74.0		
OC stage distribution: surveillance				
Local	67.0	50.0 to 98.0	Normal	Kwon et al, 2008^13^
Regional	17.0	2.0 to 42.0		
Distant	16.0	0.0 to 8.0		
OC stage distribution: RRS				
Local	80.0	60.0 to 85.0	Normal	Kwon et al, 2008^13^
Regional	5.0	4.0 to 16.0		
Distant	15.0	11.0 to 24.0		
OC 5-y relative survival				
Local	92.4	73.9 to 98.0	Beta	Howlader et al, 2019^33^
Regional	75.2	60.2 to 90.2		
Distant	29.2	23.4 to 35.0		
Utilities				
Healthy (age adjusted)	0.82 to 1.0	NA	NA	Fryback et al, 2007^35^
Hyst-BSO (premenopausal)	0.90	0.77 to 0.95	Normal	Kwon et al, 2008^13^; Anderson et al, 2006^36^; Grann et al 2010,^37^; Yamauchi et al, 2018^38^
Hyst-BSO (postmenopausal)	1.00	0.95 to 1.00		Bhattacharya et al, 2011^24^; Roberts et al, 2011^25^; Hurskainen et al, 2004^39^
Hysterectomy and bilateral salpingectomy	1.00	0.95 to 1.00		Bhattacharya et al, 2011^24^; Roberts et al, 2011^25^; Hurskainen et al, 2004^39^
Utility decrement				
Initial operation without complication	−0.025	−0.015 to −0.035	Normal	Bhattacharya et al, 2011^24^; Roberts et al, 2011^25^
Initial operation with complication	−0.0425	−0.0325 to −0.0545		Bhattacharya et al, 2011^24^; Roberts et al, 2011^25^
Surveillance	−0.0008 (one-third of 1 day)	−0.0003 to −0.001		Assumed
EC				
Local	0.83	0.73 to 0.93	Normal	Yang et al, 2001^14^
Regional	0.83	0.73 to 0.93		
Distant	0.59	0.49 to 0.69		
OC				
Local	0.75	0.65 to 0.85	Normal	Yang et al, 2001^14^
Regional	0.75	0.65 to 0.85		
Distant	0.59	0.49 to 0.69		
Cost, 2020 US $				
Initial surveillance encounter	1920	1536 to 2304	Gamma	Yang et al, 2001^14^
Subsequent surveillance encounters	1376	1101 to 1651		Yang et al, 2001^14^
Hysterectomy with or without BSO	8922	6941 to 11 586		Havrilesky et al, 2017^40^
Oophorectomy	6155	4657 to 8508		Havrilesky et al, 2017
Surgical complication	8276	6621 to 9931		Havrilesky et al, 2017
EC initial cost of care				
Local	22 821	21 707 to 23 937	Gamma	Yabroff et al, 2008^41^
Regional	42 396	38 837 to 45 955		
Distant	71 074	61 147 to 81 001		
EC continued care	1532	1355 to 1711		
EC end of life care	41 226	39 751 to 42 699		
OC initial cost of care				
Local	50 653	43 801 to 57 504	Gamma	Yabroff et al, 2008^41^
Regional	70 056	58 998 to 81 112		
Distant	97 312	92 875 to 101 750		
OC continued care	6509	5914 to 7104		
OC end of life care	83 876	80 951 to 86 799		

Abbreviations: CA-125, cancer antigen 125; CDC, Centers for Disease Control and Prevention; EC, endometrial cancer; hyst-BSO, hysterectomy with bilateral salpingo-oophorectomy; NA, not applicable; OC, ovarian cancer; RRS, risk-reducing surgical treatment; TVUS, transvaginal ultrasound.

In the hyst-BSO groups, risk of EC and OC were set to 0 after surgical treatment. In the 2-stage strategy, EC risk was set to 0 after hyst-BS at age 40 years, but patients remained at risk for OC until oophorectomy was performed at age 50 years.^26,27,28,29^ As in past LS models, we assumed that surveillance or surgical treatment would be associated with downstaging but that the downstaging benefit would be greater for EC than for OC (Table 2).^12,13^

In the surveillance groups, patients not yet diagnosed with cancer received OC screening via annual TVUS and CA-125 testing and EC screening via annual pelvic examination and endometrial biopsy. Patients were assumed to be 100% compliant with screening. Patients with a false-negative result transitioned into undetected cancer states and were diagnosed with cancer in the subsequent cycle. We assumed that anyone receiving a false-positive result would immediately undergo hyst-BSO. Individuals who received a true positive result entered stage-specific EC and OC states based on published stage distributions of patients with LS in gynecologic cancer surveillance programs.^12,13,31,32^ Probabilities of false positive, false negative, true positive, and true negative results were obtained by multiplying surveillance performance characteristics by cancer risk.

Cancer death rates were obtained from the literature, and all-cause death rates were derived from Centers for Disease Control and Prevention life tables. Current evidence suggests an increase in all-cause mortality after early menopause, although estimates vary from a 12% to an 81% increase in risk of death.^42,43,44,45^ There is also evidence that hormone replacement therapy is associated with a decrease in this risk^46^ or elimination of this risk.^43,45,47^ No increase in all-cause mortality after early, surgical menopause was reported in 2 additional studies.^48,49^ Given mixed evidence, we assumed no added risk of all-cause mortality associated with oophorectomy in our baseline model but examined potential associations with increased mortality in 1-way and threshold sensitivity analyses.

### Costs

Direct medical costs were assessed from a health care system perspective and derived from Medicare data and published analyses (eTable 2 in the Supplement). Costs were discounted at a 3% annual rate^50^ and adjusted for inflation from their initial year of publication to 2020 using the Medical Consumer Price Index.^51^ All costs are reported in US dollars.

### Quality of Life and Utilities

Quality of life (QOL) health state utility values were obtained from the literature. Utility decrements associated with surgical menopause with hormone therapy were applied to patients in the post–hyst-BSO health state until age 50 years, the mean age of menopause.^52,53^ Utility decrements associated with complication rates of surgical treatment and convalescence time were applied to the first postoperative year after RRS (eTable 3 in the Supplement).^24,25^ A utility decrement of one-third of 1 day was applied to individuals undergoing surveillance to account for time lost. All utilities were subjected to half-cycle correction and a 3% annual discount rate.^50^

### Statistical Analysis

The primary outcomes were QALYs gained and associated lifetime costs in each strategy. Accordingly, we defined the optimal strategy as that which yielded the greatest QALYs with an incremental cost-effectiveness ratio (ICER) below a willingness-to-pay (WTP) threshold of $100 000. We calculated ICERs by ordering strategies by total discounted cost and dividing the difference in costs by the difference in QALYs from the next most costly, undominated strategy. Secondary outcomes included unadjusted life-years (ie, survival), cancer incidence, and cancer mortality.

Sensitivity analyses were performed to test the association of model input uncertainty with robustness of base case results. In 1-way, deterministic sensitivity analyses, we varied each model input separately to assess the association of each variable with the ICER. To test the overall association of uncertainty in model input parameters with outcomes, we performed a probabilistic sensitivity analysis (PSA) in which we varied each input parameter simultaneously by sampling distributions across 10 000 samples. In the PSA, we determined inputs by sampling beta distributions (probabilities), gamma distributions (costs), and normal distributions (utilities).^54^ In each trial, we tested all 12 strategies for each gene, resulting in 480 000 simulations (Table 2). When plausible ranges (ie, SDs and ranges from past cost-effectiveness studies) were not available, we tested values 10% above and below the base case value for probabilities, 20% above and below the base case value for costs, and 0.10 above and below the base case estimate. If 10% above and below a base case probability value was not amenable to a beta distribution, we sampled a normal distribution with the base case value as the mean and 10% above and below the base case value as the SD.

## Results

### Base Case

The optimal strategy, yielding the highest QALYs with an ICER below a $100 000 WTP threshold, differed by gene (Table 3; eFigure 2 in the Supplement). The 2-stage approach (ie, hyst-BS at age 40 years and oophorectomy at age 50 years) was optimal for women with *MLH1* and *MSH6* variants, yielding 22.47 QALYs and 22.49 QALYs, respectively (Figure). The 2-stage approach was also cost-effective for individuals with *MLH1* and *MSH6* variants, with associated ICERs of $33 269 and $20 008 compared with hyst-BSO at age 40 years. However, the 2-stage approach, compared with hyst-BSO at age 40 years, was associated with increased cancer incidence that varied by gene variant (*MLH1*: 7.76% vs 3.84%; *MSH6:* 7.24% vs 4.52%) and increased cancer mortality that varied by gene variant (*MLH1:* 5.74% vs 2.55%; *MSH6:* 5.22% vs 2.97%) (Table 3; eFigure 3 and eTable 4 in the Supplement). Hyst-BSO at age 40 years was optimal for individuals with *MSH2* variants, associated with 22.23 QALYs and an ICER of $5180 compared with hyst-BSO at age 35 years. For individuals with *MSH2* variants, hyst-BSO at age 40 years was associated with a total cancer incidence of 4.42% and cancer mortality of 2.97%. For individuals with *PMS2* variants, hyst-BSO delayed until age 50 years was optimal, given that all other strategies were dominated. Hyst-BSO at age 50 years yielded 23.04 QALYs, a cancer incidence rate of 0.68%, and a cancer mortality rate of 0.29%. The Figure demonstrates the tradeoff between cancer incidence and QALYs by gene and strategy. Comparisons between modeled and published cancer incidence rates supported the validity of the model (eFigures 4 and 5 in the Supplement). Results for all strategies are detailed in eTable 4 in the Supplement.

**Table 3.** Primary and Secondary Outcomes by Genotype

Strategy	QALYs, No.	Life-years, No.	EC incidence (death), %	OC incidence (death), %	Total cost, $	ICER, $
*MLH1*						
Hyst-BSO: 35	22.09	47.01	0.95 (0.46)	1.00 (0.82)	8642	0
Hyst-BSO: 40	22.27	46.68	1.87 (0.89)	1.97 (1.66)	9261	3520
2-Stage approach^a^^,^^b^	22.47	45.97	1.87 (0.89)	5.89 (4.85)	15 935	33 269
*MSH2*						
Hyst-BSO: 35	22.07	46.96	1.15 (0.55)	1.10 (0.90)	8879	0
Hyst-BSO: 40^a^	22.23	46.58	2.26 (1.08)	2.16 (1.82)	9692	5180
*MSH6*						
Hyst-BSO: 35	22.06	46.95	1.15 (0.55)	1.15 (0.94)	8950	0
Hyst-BSO: 40	22.22	46.55	2.26 (1.07)	2.26 (1.90)	9823	5726
2-Stage approach^a^	22.49	46.06	2.26 (1.07)	4.98 (4.15)	15 303	20 008
*PMS2*						
Hyst-BSO: 40	22.56	47.38	0.19 (0.09)	0 (0)	5888	Dominated
Hyst-BSO: 50^a^	23.04	47.36	0.68 (0.29)	0 (0)	4470	0

Abbreviations: hyst-BSO, hysterectomy with bilateral salpingo-oophorectomy; ICER, incremental cost-effectiveness ratio; QALYs, quality-adjusted life-years.

^a^ This was an optimal strategy.

^b^ The 2-stage approach was hysterectomy with bilateral salpingectomy at age 40 years with oophorectomy at age 50 years.

**Figure.**
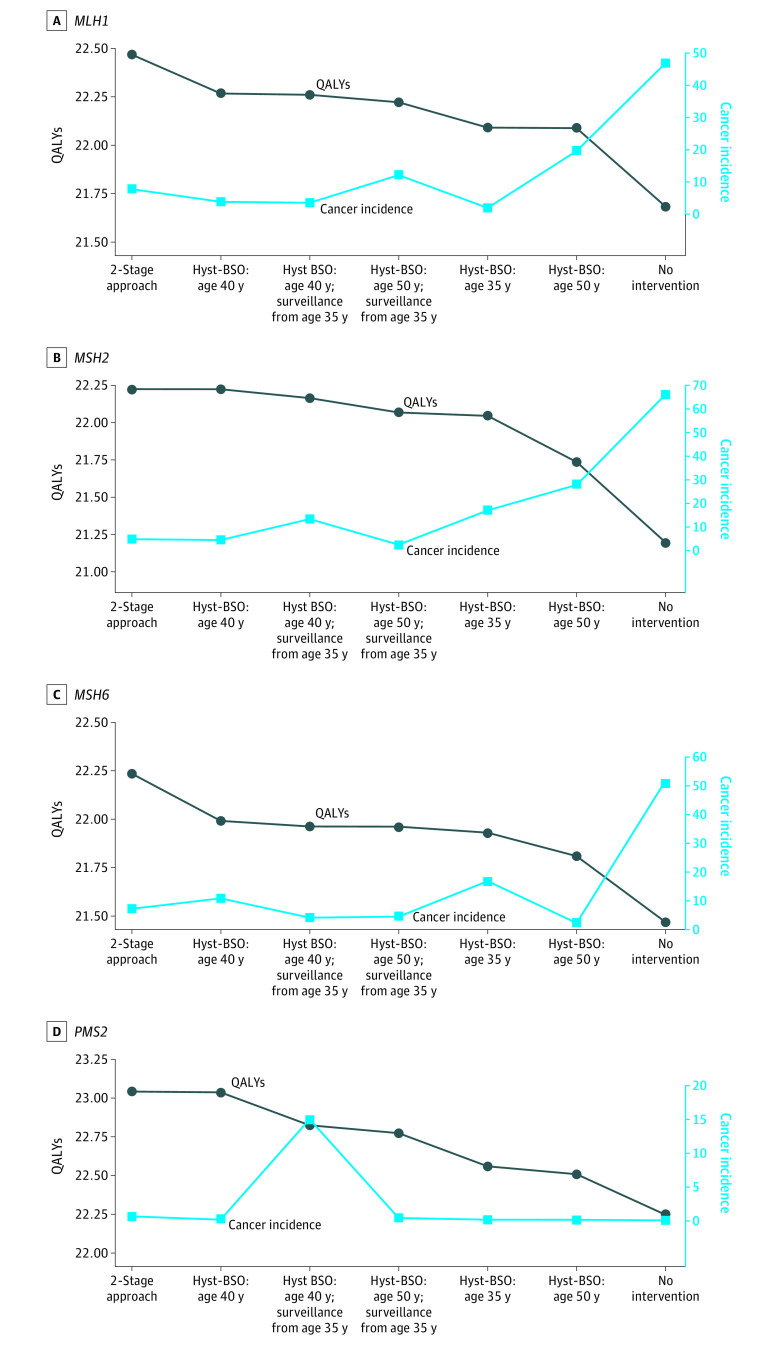
Quality-Adjusted Life-Years (QALYs) and Cancer Incidence Tradeoff by Strategy and Gene Hyst-BSO indicates hysterectomy and bilateral salpingo-oophorectomy.

### 1-Way Sensitivity Analysis

For *MLH1, MSH2,* and *MSH6* variants*,* the variables associated with the greatest difference in outcomes included the risk of all-cause mortality associated with oophorectomy, disutility of early menopause, disutility of hysterectomy, and lifetime OC risk (eFigure 6 in the Supplement). We performed threshold analyses on these variables and on the association of hyst-BS with OC risk (Table 4; eFigures 7, 8, 9, and 10 in the Supplement).

**Table 4.** Threshold Analyses

Variable	Variable range	Optimal strategy below $100 000 WTP
Utility of early menopause^b^		
* MLH1*	0.86-0.92	2-Stage approach^a^
	0.93-0.95	Hyst-BSO: 40
* MSH2*	0.86	2-Stage approach
	0.87-0.94	Hyst-BSO: 40
	0.95	Hyst-BSO: 35
* MSH6*	0.86-0.94	2-Stage approach
	0.95	Hyst-BSO: 35
* PMS2*	0.86-0.95	Hyst-BSO: 50
Utility of hysterectomy (or hyst-BSO without early menopause)^b^		
* MLH1*	0.95-0.96	Hyst-BSO: 40
	0.97-1	2-Stage approach
* MSH2*	0.95-1.00	Hyst-BSO: 40
* MSH6*	0.95-1.00	2-Stage approach
* PMS2*	0.95-0.96	No intervention
	0.97-1	Hyst-BSO: 50
Risk of OC development after hysterectomy and salpingectomy, RR		
* MLH1*	0.75 (greatest benefit)-1.00 (no benefit)	2-Stage approach
* MSH2*	0.75-1.00	Hyst-BSO: 40
* MSH6*	0.75-1.00	2-Stage approach
* PMS2*	0.75-1.00	Hyst-BSO: 50
Lifetime OC risk, %		
* MLH1*	8.4-19.7	2-Stage approach
* MSH2*	11.8	2-Stage approach
	12.9-31.2	Hyst-BSO: 40
* MSH6*	4.7-19.6	2-Stage approach
	23.2-33.9	Hyst-BSO: 35, survey: 30
* PMS2*	0.06-47.4	Hyst-BSO: 50
Risk of all-cause mortality after oophorectomy, RR		
* MLH1*	1.0 (no added risk)-1.4	2-Stage approach
* MSH2*	1.0-1.24	Hyst-BSO: 40
	1.32-1.4	2-Stage approach
* MSH6*	1.0-1.4	2-Stage approach
* PMS2*	1.0-1.4	Hyst-BSO: 50

Abbreviations: hyst-BSO, hysterectomy with bilateral salpingo-oophorectomy; OC, ovarian cancer; RR, risk ratio; WTP, willingness-to-pay threshold.

^a^ The 2-stage approach was hysterectomy with bilateral salpingectomy at age 40 years with oophorectomy at age 50 years.

^b^ Utility values ranged from 0 (death) to 1 perfect health).

For the utility of early menopause, the 2-stage approach remained optimal for individuals with *MLH1* for values below 0.93. At or above 0.93, hyst-BSO at age 40 years became optimal. For individuals with *MSH2*, hyst-BSO at age 40 years was optimal for early menopause utility values of 0.87 to 0.94. For values less than 0.87, the 2-stage approach was optimal for individuals with *MSH2* variants, and greater than 0.94, hyst-BSO at age 35 years was optimal. For individuals with *MSH6* variants, the 2-stage approach remained optimal for early menopause utility values less than 0.95 but hyst-BSO at age 35 years was cost-effective for values greater than 0.95. Hyst-BSO at age 50 years was optimal for individuals with *PMS2* variants at all early menopause utility values tested.

We assumed no added disutility of hysterectomy (or hyst-BSO at age 50 years, which we assumed would be after menopause) in the base case. Testing this assumption in sensitivity analyses, the optimal strategy did not change for individuals with *MSH2* or *MSH6* variants*.* For individuals with *MLH1* variants, hyst-BSO at age 40 years was favored for posthysterectomy utility values less than 0.97 (base case: 1.0). For individuals with *PMS2* variants, no intervention (ie, natural history without RRS) was favored if postsurgical utility values were less than 0.97.

The lifetime risk of OC development was not associated with a change in the optimal strategy for individuals with *MLH1* or *PMS2* variants. The 2-stage approach was optimal for individuals with *MSH2* variants when the risk of OC development was at the lower end of estimated lifetime OC risk. As OC risk increased for *MSH6,* more aggressive strategies, such as hyst-BSO at age 35 with or without surveillance, were favored (OC risk 4.7%-19.6%: 2-stage approach favored; OC risk 23.2%-33.9%: hyst-BSO at age 35 years favored) (Table 4).

Varying the decrease in risk of OC development associated with hyst-BS was not associated with changes in outcomes for any genes (Table 4). However, for *MLH1* and *MSH6,* the incremental cost-effectiveness of the 2-stage approach compared with hyst-BSO at age 40 years decreased as the risk reduction approached 0% (the value assumed in the base case) (eFigure 7 in the Supplement).

Added risk of all-cause mortality associated with oophorectomy was not associated with a significant change in the overall outcomes for individuals with *MLH1, MSH6,* or *PMS2* variants (Table 4). However, for *MLH1* and *MSH6,* as the added risk of all-cause mortality associated with oophorectomy increased, the incremental cost-effectiveness of the 2-stage approach increased (eFigure 8 in the Supplement). In other words, increasing the risk of all-cause mortality associated with oophorectomy was associated with more favorable outcomes for the 2-stage approach compared with hyst-BSO at age 40 years. When the added risk of mortality associated with early menopause exceeded 24%, the 2-stage approach became optimal for individuals with *MSH2* variants (eFigure 10 in the Supplement)*.* The added risk of all-cause mortality was not associated with a change in outcomes for *PMS2* (Table 4; eFigure 8 in the Supplement)*.*

### PSA

Results from PSA are shown in the cost-effectiveness acceptability curve, which displays the likelihood that a strategy was optimal at varying WTP thresholds (eFigure 11 in the Supplement). The base case results were robust to uncertainty in model input parameters for all LS subtypes. At a $100 000 WTP, the 2-stage approach was optimal in 8418 of 10 000 iterations (84.2%) for *MLH1* and 7103 of 10 000 iterations (71.0%) for *MSH6*. Hyst-BSO at age 40 years was optimal for *MSH2* in 8620 of 10 000 iterations (86.2%), while the 2-stage surgical approach was optimal in 1216 iterations (12.2%) and hyst-BSO at age 35 years was optimal in 163 iterations (1.6%). Additionally, hyst-BSO at age 50 years was optimal for *PMS2* in 9162 of 10 000 iterations (91.6%). The outcomes for the base case optimal strategies in the PSA are displayed in eTable 5 in the Supplement.

## Discussion

This economic evaluation’s findings suggest that genotype-specific strategies for gynecologic cancer screening and risk reduction among women with LS may be cost-effective and associated with improved quality of life. We found that a novel 2-stage surgical approach with hysterectomy and bilateral salpingectomy at age 40 years and oophorectomy delayed until age 50 years was optimal for individuals with *MLH1* and *MSH6* variants, while surgical treatment at age 40 years was optimal for individuals with *MSH2* variants and delay in surgical treatment until age 50 years was optimal for individuals with *PMS2* variants. All together, these results suggest that a one size fits all approach to gynecologic cancer risk management may not be supported for individuals with LS.

Although hyst-BSO upon completion of childbearing remains the recommended risk-reducing measure for OC, some patients with LS may be hesitant to pursue this option owing to the association of early menopause with detrimental QOL outcomes,^55,56,57^ including cardiovascular^58^ and musculoskeletal health outcomes.^59^ Because gynecologic surveillance has not been well-supported, consideration of alternative options, such as delayed oophorectomy, may be associated with benefits for patients who are reluctant to undergo BSO before natural menopause. Past gene-agnostic simulation modeling analyses^12,13,14^ have similarly found that RRS is preferred over gynecological surveillance. Furthermore, surveillance strategies for gynecologic cancers lack specificity and are associated with significant false positive testing rates that lead to downstream testing and patient anxiety. In our analysis, the substantial differences in cancer risk associated with individual LS genes suggest that mutation-specific prophylaxis strategies may be considered.

The observation that many OCs arise from the distal fallopian tube has led to increased use of opportunistic salpingectomy among women undergoing gynecologic surgical treatment and prompted interest in staged surgical procedures for women at increased risk for OC. In this approach, salpingectomy is initially performed, with oophorectomy performed years afterward to mitigate adverse outcomes of hormonal deprivation and menopause among young women. Trials of staged surgical prophylaxis are currently ongoing for individuals with *BRCA* variants,^15,26,27,28,29,30^ and the generalizability of these findings to women with LS remains unknown.

Although the 2-stage surgical approach was the most cost-effective strategy for individuals with *MLH1* and *MSH6* variants, it was associated with an increased risk of cancer compared with risk-reducing hyst-BSO at age 40 years. This potential approach requires additional study and may provide an alternative cancer risk–reduction option for patients concerned about surgical menopause.

Among all women with LS, those with *MSH2* variants have the highest risk for gynecologic cancer. In our study, hyst-BSO at age 40 years was optimal in 86% of trials in the PSA, while the 2-stage surgical approach was preferred in 12% of iterations and hyst-BSO at age 35 years was optimal in 2% of trials. Our results suggest that conventional management with hyst-BSO at age 40 years should remain the preferred approach.

In contrast, individuals with *PMS2* variants have the lowest risk for gynecologic cancer compared with individuals with other LS MMR gene variants. Gynecologic malignant neoplasms manifest later among individuals with *PMS2* variants, with an incidence of more than 1% before age 50 years.^3,4^ Our findings suggest that current guidelines recommending prophylactic surgical treatment at age 40 years potentially increase risk of negative outcomes associated with premature menopause with minimal value. In our model, delay of surgical treatment until age 50 years had the most favorable outcome for individuals with *PMS2* variants.

### Limitations

While our study benefits from the analysis of a variety of prophylactic strategies, we recognize that the study has several limitations. As with all computational simulation studies, our study relied on the quality of data used for parameter inputs. To account for potential uncertainties owing to limited data and simplifying assumptions, we conducted extensive sensitivity analyses. Similarly, we lacked data on individual or family history of LS-associated cancers that are associated with cancer risk. If these data were to become available, microsimulation studies would do well to investigate the association of specific family histories with the cost-effectiveness of current and potential risk-reduction strategies. Additionally, we did not account for other LS-associated cancers, given that our primary focus was gynecologic cancer.

## Conclusions

Our study findings support a gene-specific approach for gynecologic cancer risk reduction for individuals with LS. These findings suggest that individuals with *PMS2* variants may benefit from less aggressive treatment with hysterectomy with BSO delayed until age 50 years and individuals with *MSH2* variants may benefit from earlier hyst-BSO at age 40 years. For individuals with *MLH1* and *MSH6* variants, our findings suggest that the novel, 2-stage RRS approach is a potential cost-effective alternative for those wishing to delay surgically induced menopause. Decision-making regarding risk-reduction options for the prevention of gynecological cancers among individuals with LS warrants further study, including the association of patient preferences and perception of cancer risk with decision-making outcomes.

## Notes

Supplement. **eFigure 1.** Model Overview **eTable 1.** Cancer Risks by Gene and Age **eTable 2.** Detailed Cost Calculations **eTable 3.** Detailed Utility Calculations **eFigure 2.** Base Case Results of Risk-Reducing Surgical Approaches **eFigure 3.** Cancer Incidence and Mortality by Gene and Strategy **eFigure 4.** Calibration of Endometrial Cancer Incidence by Gene **eFigure 5.** Calibration of Ovarian Cancer Incidence by Gene **eTable 4.** Full Model Outputs for Base Case Analysis **eFigure 6.** 1-Way Sensitivity Analysis **eFigure 7.** Threshold Analysis: Risk Ratio for Ovarian Cancer Development Postsalpingectomy **eFigure 8.** Threshold Analysis: Risk Ratio for All-Cause Mortality Postoophorectomy **eFigure 9. **Threshold Analysis: Utility of Postoperation Status (Without Menopause) **eFigure 10.** Threshold Analysis: Utility of Early Menopause **eFigure 11.** Cost-Effectiveness Acceptability Results for Probabilistic Sensitivity Analysis **eTable 5.** Variation in Base Case Results From 10 000 Probabilistic Sensitivity Analysis Samples
eReferences.
