# Supplementary material for: Cost-effectiveness Analysis of Genotype-Specific Surveillance and Preventive Strategies for Gynecologic Cancers Among Women With Lynch Syndrome
Source: JAMA Netw Open. 2021 Sep 9;4(9):e2123616. doi: 10.1001/jamanetworkopen.2021.23616 (PMC8430458; doi:10.1001/jamanetworkopen.2021.23616)
Supplement: Supplement. — eFigure 1. Model Overview eTable 1. Cancer Risks by Gene and Age eTable 2. Detailed Cost Calculations eTable 3. Detailed Utility Calculations eFigure 2. Base Case Results of Risk-Reducing Surgical Approaches eFigure 3. Cancer Incidence and Mortality by Gene and Strategy eFigure 4. Calibration of Endometrial Cancer Incidence by Gene eFigure 5. Calibration of Ovarian Cancer Incidence by Gene eTable 4. Full Model Outputs for Base Case Analysis eFigure 6. 1-Way Sensitivity Analysis eFigure 7. Threshold Analysis: Risk Ratio for Ovarian Cancer Development Postsalpingectomy eFigure 8. Threshold Analysis: Risk Ratio for All-Cause Mortality Postoophorectomy eFigure 9. Threshold Analysis: Utility of Postoperation Status (Without Menopause) eFigure 10. Threshold Analysis: Utility of Early Menopause eFigure 11. Cost-Effectiveness Acceptability Results for Probabilistic Sensitivity Analysis eTable 5. Variation in Base Case Results From 10 000 Probabilistic Sensitivity Analysis Samples eReferences. [file jamanetwopen-e2123616-s001.pdf]

Supplemental Online Content

Wright JD, Silver ER, Tan SX, Hur C, Kastrinos F. Cost-effectiveness analysis of genotype-specific surveillance and preventive strategies for gynecologic cancers among women with Lynch syndrome. *JAMA Netw Open*. 2021;4(9):e2123616. doi:10.1001/jamanetworkopen.2021.23616

eFigure 1. Model Overview

eTable 1. Cancer Risks by Gene and Age

eTable 2. Detailed Cost Calculations

eTable 3. Detailed Utility Calculations

eFigure 2. Base Case Results of Risk-Reducing Surgical Approaches

eFigure 3. Cancer Incidence and Mortality by Gene and Strategy

eFigure 4. Calibration of Endometrial Cancer Incidence by Gene

eFigure 5. Calibration of Ovarian Cancer Incidence by Gene

eTable 4. Full Model Outputs for Base Case Analysis

eFigure 6. 1-Way Sensitivity Analysis

eFigure 7. Threshold Analysis: Risk Ratio for Ovarian Cancer Development Postsalpingectomy

eFigure 8. Threshold Analysis: Risk Ratio for All-Cause Mortality Postoophorectomy

eFigure 9. Threshold Analysis: Utility of Postoperation Status (Without Menopause)

eFigure 10. Threshold Analysis: Utility of Early Menopause

eFigure 11. Cost-Effectiveness Acceptability Results for Probabilistic Sensitivity Analysis

© 2021 Wright JD et al. *JAMA Network Open.*

eTable 5. Variation in Base Case Results From 10 000 Probabilistic Sensitivity Analysis Samples

eReferences.

This supplemental material has been provided by the authors to give readers additional information about their work.

© 2021 Wright JD et al. *JAMA Network Open.*

eFigure 1. Model Overview
Surveillance and Hyst-BSO strategies are shown in top panel, and the two-stage approach with Hyst-BS with delayed oophorectomy are shown in bottom panel.

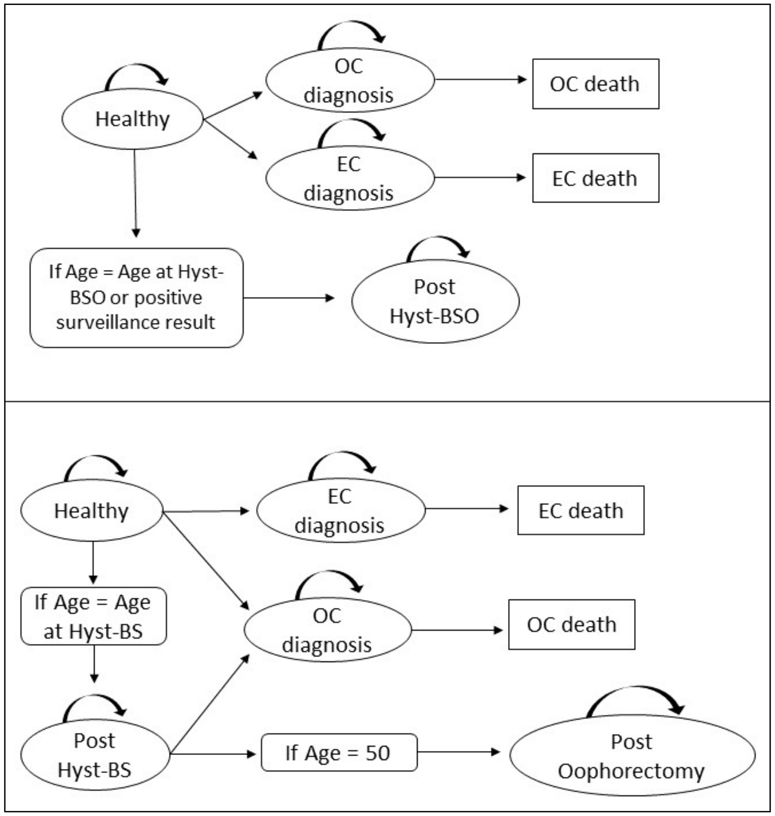

© 2021 Wright JD et al. *JAMA Network Open.*

eTable 1. Cancer Risks by Gene and Age

Gene	Age	EC Risk (Range)	OC Risk (Range)	Source
MLH1				
	30	0.00% (0.00% - 2.6%)	0.00% (0.00% - 2.6%)	[1]
	40	1.90% (0.80% - 4.8%)	2.0% (0.9% - 5.0%)	[1]
	50	14.7% (11.00% - 19.50%)	6.1% (3.8% - 9.8%)	[1]
	60	27.3% (22.10% - 33.60%)	10.1% (6.8% - 15.1%)	[1]
	70	35.20% (28.80% - 43.40%)	11.0% (7.4% - 17.1%)	[1]
	75	37% (30.1% - 46.5%)	12.0% (8.4% - 18.1%)	Imputed
MSH2				
	30	0.0% (0.0% - 3.3%)	0.0% (0.0% - 3.3%)	[1]
	40	2.3% (0.90% - 6.2%)	2.2% (0.90% - 6.0%)	[1]
	50	17.5% (12.8% - 23.7%)	10.5% (6.9% - 15.7%)	[1]
	60	38.0% (30.9% - 46.2%)	12.6% (8.5% - 18.4%)	[1]
	70	46.5% (38.3% - 56.3%)	-	[1]
	75	48.9% (40.2% - 60.7%)	17.4% (11.8% - 31.2%)	[1]
MSH6				
	30	0.0% (0.0% - 22.6%)	0.0% (0.0% - 0.0%)	[1]
	40	2.3% (0.4% - 24.6%)	2.3% (0.4% - 12.3%)	[1]
	50	12.6% (6.3% - 33.1%)	-	[1]
	55	-	6.6% (2.1% - 22.6%)	[1]
	60	28.3% (18.2% - 46.5%)	-	[1]
	70	41.1% (28.6% - 57.9%)	10.8% (3.7% - 32.9%)	[1]
	75	42.1% (29.6% - 58.9%)	11.8% (4.7% - 33.9%)	Imputed
PMS2				
	30	0.0% (0.0% - 0.0%)	0.0% (0.0% - 41.4%)	[1,2]
	40	0.02% (0.0% - 0.04%)	0.0% (0.0% - 42.0%)	[1,2]
	50	0.07% (0.0% - 1.4%)	0.0% (0.0% - 42.7%)	[1,2]
	60	7.1% (0.5% - 13.7%)	3.0% (0.1% - 43.3%)	[1,2]
	70	11.78% (3.6% - 20.0%)	-	[1,2]
	75	12.78% (4.6% - 21.0%	4.0% (0.1% - 43.4%)	Imputed

© 2021 Wright JD et al. *JAMA Network Open.*

eTable 2. Detailed Cost Calculations

Variable	Cost	Year of Cost	Cumulative Inflation Rate	2020 Cost	Source
Office visit—first gynecologic exam	$596.00	2010	33.58%	796.1547	3
Office visit—subsequent gynecologic exams	$189.00	2010	33.58%	252.4719	3
CA125 testing	$78.00	2010	33.58%	104.1947	3
Transvaginal ultrasound	$113.00	2010	33.58%	150.9488	3
Endometrial biopsy	$118.00	2010	33.58%	157.6279	3
Pathology evaluation for endometrial biopsy	$532.00	2010	33.58%	710.6616	3
Total cost of initial surveillance encounter				1919.588	Calculated
Total cost of subsequent surveillance encounters				1375.905	Calculated
Hysterectomy +/- BSO	$7,682.00	2015	16.14%	8921.568	4
BSO	$5300.00	2015	16.14%	6155.208	4
Surgical complications					
UTI	$5,436.00	2015	16.14%	6313.153	4
Wound infection	$8,658.00	2015	16.14%	10055.05	4
Vaginal cuff infection	$8,658.00	2015	16.14%	10055.05	4
Venous thromboembolism	$5,753.00	2015	16.14%	6681.304	4
Average total complication cost				8276.142	Calculated
Initial EC care					
Local	$13,646.00	2004	67.24%	$22,821.16	5
Regional	$25,351.00	2004	67.24%	$42,396.25	5
Distant	$42,499.00	2004	67.24%	$71,074.05	5

© 2021 Wright JD et al. *JAMA Network Open.*

Continuing EC care	$916.00	2004	67.24%	1531.891	5
End of life EC care	$24,651.00	2004	67.24%	41225.59	5
Initial OC care					
Local	$30,288.00	2004	67.24%	50652.74	5
Regional	$41,890.00	2004	67.24%	70055.58	5
Distant	$58,188.00	2004	67.24%	97311.87	5
Continuing OC care	$3,892.00	2004	67.24%	6508.864	5
End of life OC care	$50,154.00	2004	67.24%	83876.04	5

Note: original prices were adjusted for inflation using the Consumer Price Index for medical care: https://www.halfhill.com/inflation_js.html

eTable 3. Detailed Utility Calculations

	Utility	Percent of first post-op month to which utility value applies	Reference
Convalescence	0.74	75%	[6,7]
Operation	0.56	25%	[48][6,7]
First month utility (weighted average)	0.695	100%	[6,7]
First month disutility	-0.305		
**Disutility as a fraction of the year**	**-0.025**		
Complication	0.49	100%	
First month disutility	-0.305		
**Disutility as a fraction of the year**	**-0.0425**		

© 2021 Wright JD et al. *JAMA Network Open.*

eFigure 2. Base Case Results of Risk-Reducing Surgical Approaches

Incremental cost-effectiveness ratios (ICERs) are represented along dashed lines. Ooph.: oophorectomy; Hyst-BSO: hysterectomy with bilateral salpingo-oophorectomy. QALYs: quality-adjusted life-years.

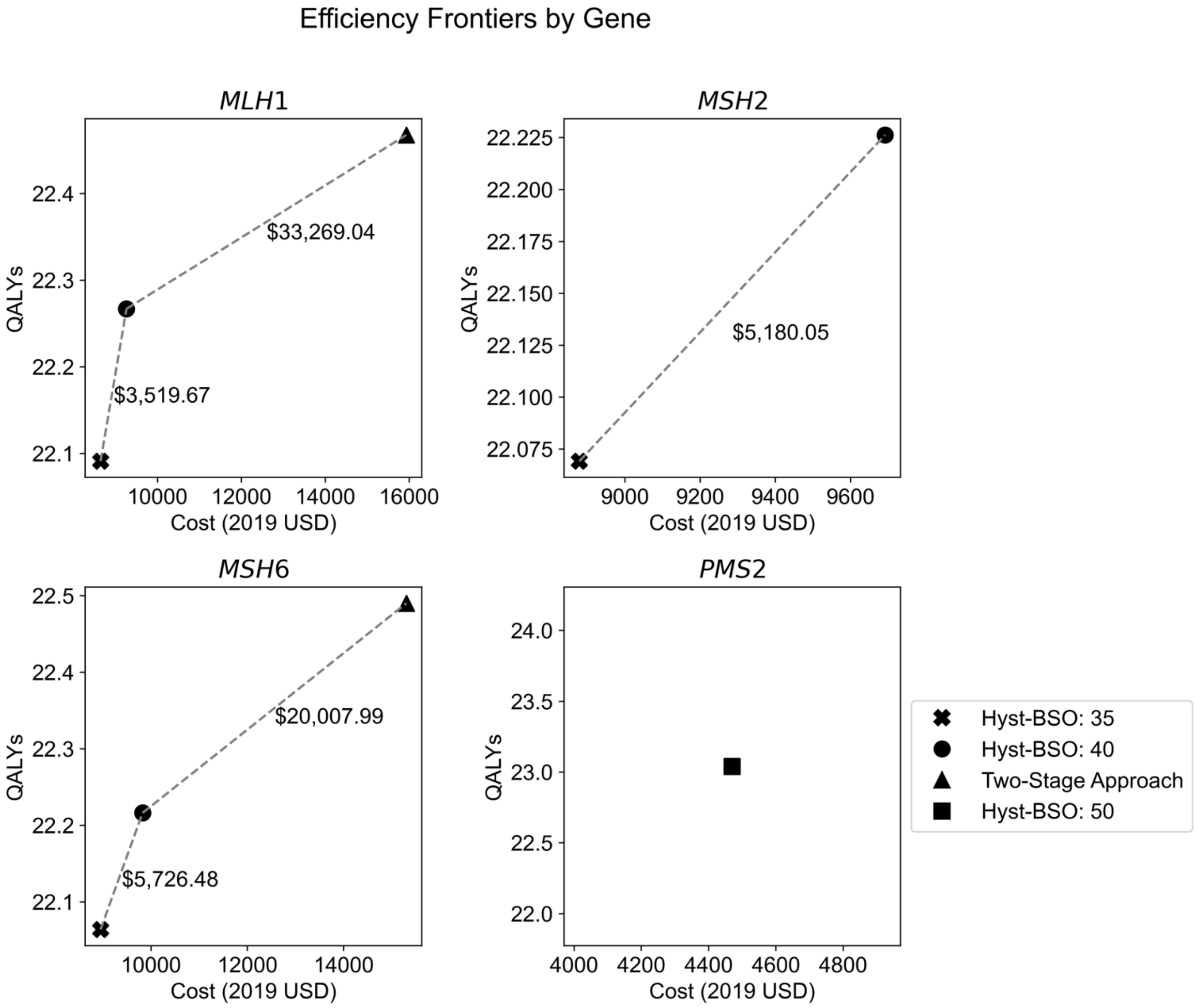

© 2021 Wright JD et al. *JAMA Network Open.*

eFigure 3. Cancer Incidence and Mortality by Gene and Strategy

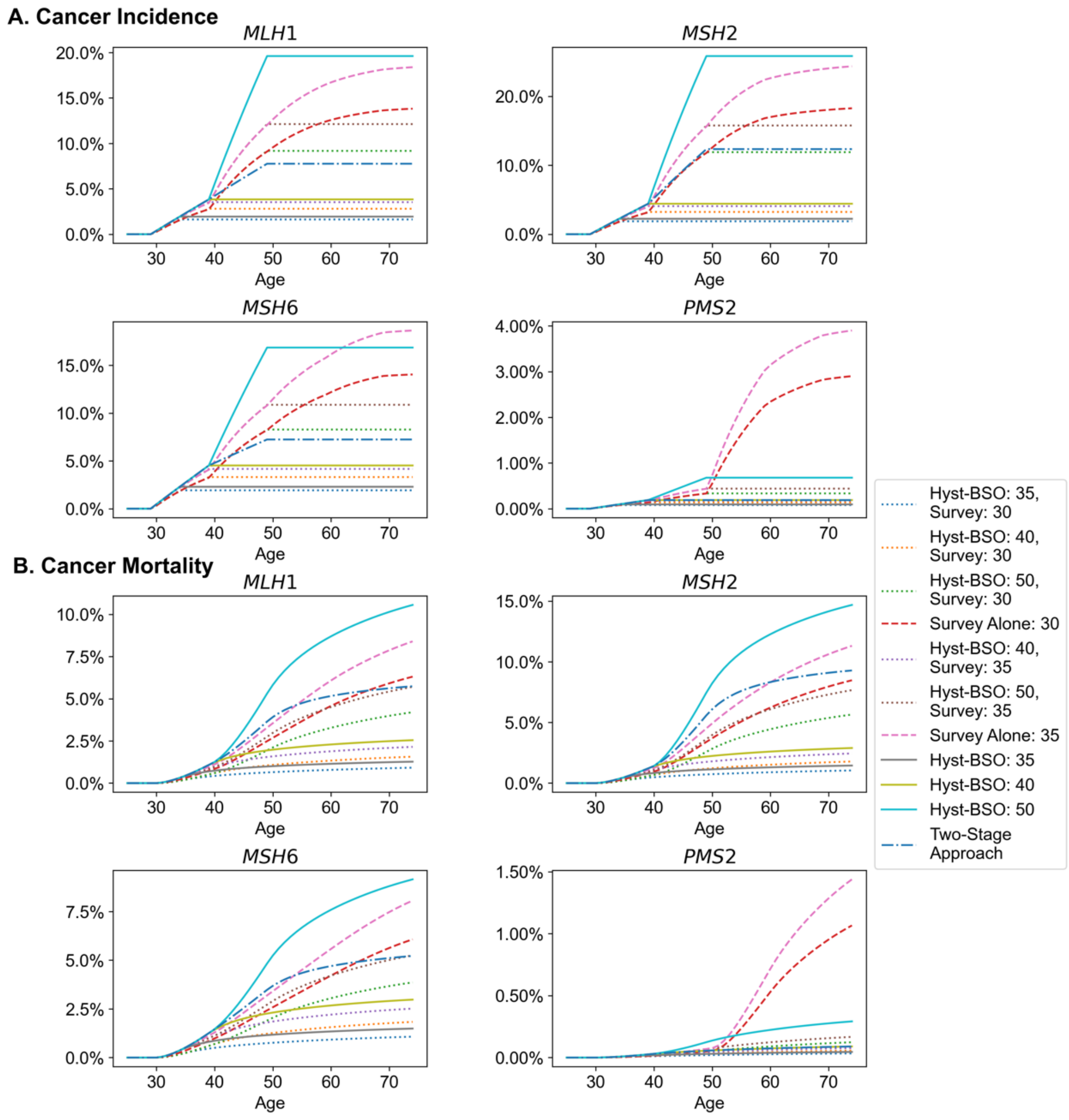

© 2021 Wright JD et al. *JAMA Network Open.*

eFigure 4. Calibration of Endometrial Cancer Incidence by Gene

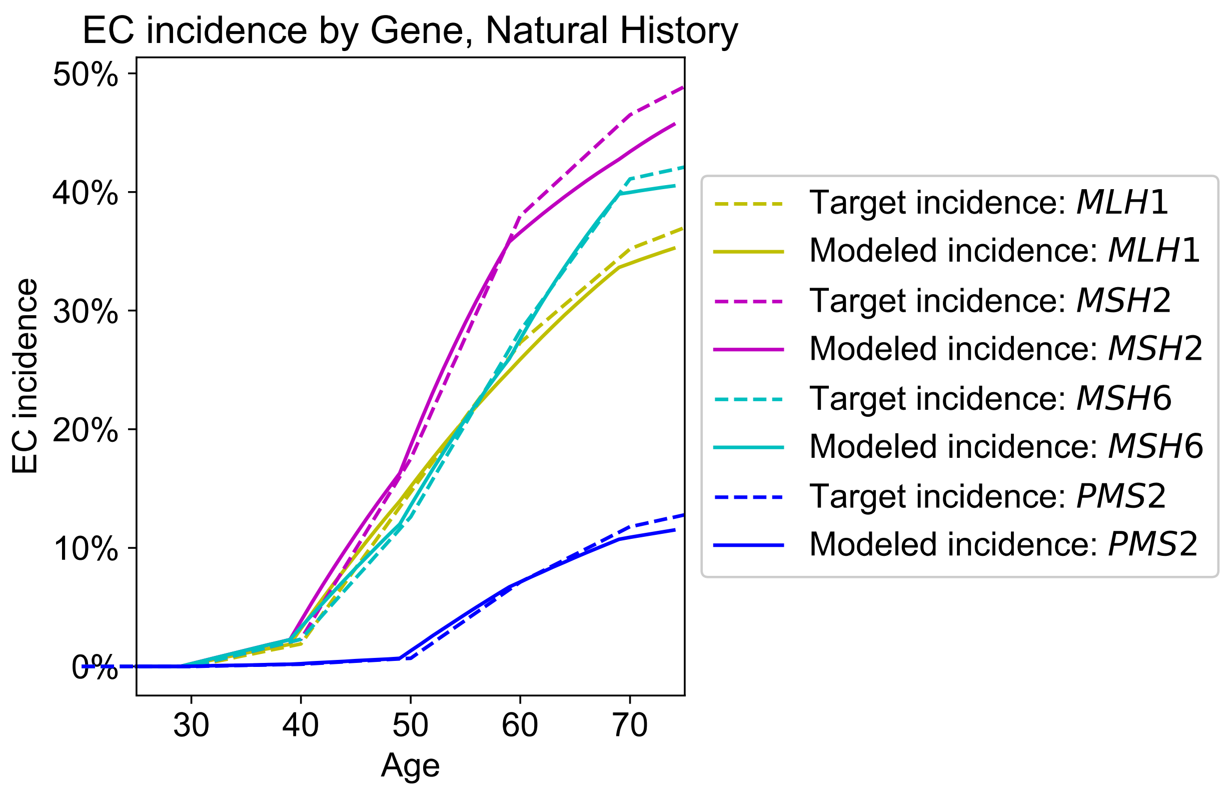

© 2021 Wright JD et al. *JAMA Network Open.*

eFigure 5. Calibration of Ovarian Cancer Incidence by Gene

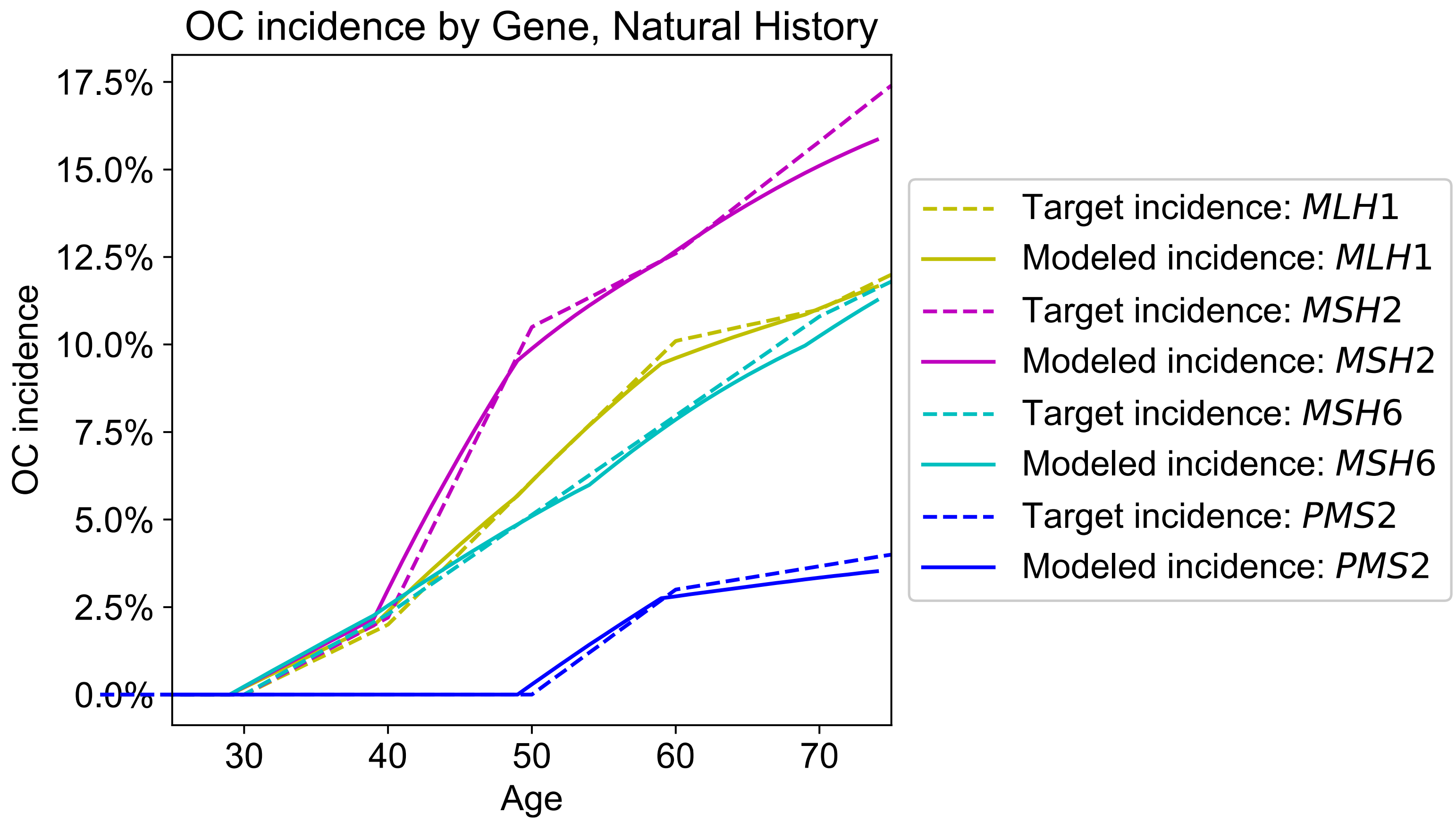

© 2021 Wright JD et al. *JAMA Network Open.*

eTable 4. Full Model Outputs for Base Case Analysis

Strategy	QALYs	Life-Years	OC Incidence (Mortality)	EC Incidence (Mortality)	Total Cost	ICER
MLH1						
Hyst-BSO: 35, Survey: 30	22.07	47.14	0.84% (0.58%)	0.79% (0.34%)	$13,583.62	Dominated
Hyst-BSO: 40, Survey: 30	22.2	46.99	1.44% (1.01%)	1.37% (0.56%)	$16,840.25	Dominated
Hyst-BSO: 50, Survey: 30	22.17	46.5	2.94% (1.98%)	6.24% (2.23%)	$21,811.40	Dominated
Survey Alone: 30	22.04	46.14	3.99% (3.27%)	9.83% (3.04%)	$24,006.84	Dominated
Hyst-BSO: 40, Survey: 35	22.26	46.8	1.81% (1.39%)	1.72% (0.76%)	$13,406.50	Dominated
Hyst-BSO: 50, Survey: 35	22.22	46.15	3.83% (2.70%)	8.30% (3.01%)	$20,113.18	Dominated
Survey Alone: 35	22.08	45.73	5.25% (4.30%)	13.14% (4.10%)	$23,072.17	Dominated
Hyst-BSO: 35	22.09	47.01	1.00% (0.82%)	0.95% (0.46%)	$8,641.85	$0.00
Hyst-BSO: 40	22.27	46.68	1.97% (1.66%)	1.87% (0.89%)	$9,260.78	$3,519.67
Hyst-BSO: 50	22.09	45.07	5.68% (4.69%)	13.94% (5.87%)	$15,060.36	Dominated
No Intervention	21.68	43.97	11.66% (8.82%)	35.25% (11.83%)	$19,436.89	Dominated
Two-Stage Approach	22.47	45.97	5.89% (4.85%)	1.87% (0.89%)	$15,934.97	$33,269.04
MSH2						
Hyst-BSO: 35, Survey: 30	22.06	47.11	0.92% (0.64%)	0.96% (0.41%)	$13,771.38	Dominated
Hyst-BSO: 40, Survey: 30	22.17	46.93	1.58% (1.11%)	1.66% (0.68%)	$17,131.12	Dominated
Hyst-BSO: 50, Survey: 30	22.04	46.2	4.57% (3.03%)	7.33% (2.62%)	$23,660.16	Dominated
Survey Alone: 30	21.87	45.7	5.56% (4.57%)	12.70% (3.90%)	$26,022.10	Dominated
Hyst-BSO: 40, Survey: 35	22.23	46.72	1.99% (1.53%)	2.08% (0.92%)	$13,781.50	Dominated
Hyst-BSO: 50, Survey: 35	22.05	45.74	6.02% (4.12%)	9.74% (3.54%)	$22,590.69	Dominated
Survey Alone: 35	21.84	45.14	7.35% (6.04%)	16.98% (5.27%)	$25,774.30	Dominated

© 2021 Wright JD et al. *JAMA Network Open.*

Hyst-BSO: 35	22.07	46.96	1.10% (0.90%)	1.15% (0.55%)	$8,878.52	$0.00
Hyst-BSO: 40	22.23	46.58	2.16% (1.82%)	2.26% (1.08%)	$9,692.02	$5,180.05
Hyst-BSO: 50	21.74	44.15	9.54% (7.81%)	16.28% (6.87%)	$19,686.73	Dominated
No Intervention	21.19	42.72	15.85% (12.09%)	45.70% (15.48%)	$26,199.05	Dominated
Two-Stage Approach	22.17	45.16	10.08% (8.21%)	2.26% (1.08%)	$20,251.24	Dominated
MSH6						
Hyst-BSO: 35, Survey: 30	22.05	47.1	0.96% (0.67%)	0.96% (0.41%)	$13,830.38	Dominated
Hyst-BSO: 40, Survey: 30	22.17	46.92	1.65% (1.16%)	1.65% (0.68%)	$17,224.13	Dominated
Hyst-BSO: 50, Survey: 30	22.19	46.55	2.70% (1.84%)	5.58% (2.02%)	$21,526.24	Dominated
Survey Alone: 30	22.06	46.19	3.65% (2.97%)	10.39% (3.10%)	$23,786.08	Dominated
Hyst-BSO: 40, Survey: 35	22.22	46.7	2.08% (1.60%)	2.08% (0.92%)	$13,899.06	Dominated
Hyst-BSO: 50, Survey: 35	22.25	46.2	3.49% (2.52%)	7.38% (2.73%)	$19,703.67	Dominated
Survey Alone: 35	22.1	45.8	4.78% (3.88%)	13.87% (4.19%)	$22,749.39	Dominated
Hyst-BSO: 35	22.06	46.95	1.15% (0.94%)	1.15% (0.55%)	$8,950.35	$0.00
Hyst-BSO: 40	22.22	46.55	2.26% (1.90%)	2.26% (1.07%)	$9,823.02	$5,726.48
Hyst-BSO: 50	22.19	45.32	4.86% (4.06%)	12.00% (5.10%)	$13,842.00	Dominated
No Intervention	21.72	44.11	11.27% (8.07%)	40.51% (13.19%)	$19,308.78	Dominated
Two-Stage Approach	22.49	46.06	4.98% (4.15%)	2.26% (1.07%)	$15,302.99	$20,007.99
PMS2						
Hyst-BSO: 35, Survey: 30	22.18	47.39	0.00% (0.00%)	0.08% (0.03%)	$12,108.27	Dominated
Hyst-BSO: 40, Survey: 30	22.37	47.39	0.00% (0.00%)	0.14% (0.06%)	$14,515.22	Dominated
Hyst-BSO: 50, Survey: 30	22.57	47.38	0.00% (0.00%)	0.33% (0.12%)	$17,024.22	Dominated
Survey Alone: 30	22.53	47.27	0.69% (0.51%)	2.21% (0.55%)	$18,988.75	Dominated
Hyst-BSO: 40, Survey: 35	22.51	47.38	0.00% (0.00%)	0.17% (0.08%)	$10,434.89	Dominated

© 2021 Wright JD et al. *JAMA Network Open.*

Hyst-BSO: 50, Survey: 35	22.77	47.38	0.00% (0.00%)	0.44% (0.17%)	$13,818.31	Dominated
Survey Alone: 35	22.71	47.23	0.93% (0.69%)	2.97% (0.74%)	$16,467.51	Dominated
Hyst-BSO: 35	22.25	47.38	0.00% (0.00%)	0.09% (0.05%)	$6,810.95	Dominated
Hyst-BSO: 40	22.56	47.38	0.00% (0.00%)	0.19% (0.09%)	$5,888.41	Dominated
Hyst-BSO: 50	23.04	47.36	0.00% (0.00%)	0.68% (0.29%)	$4,470.24	$0.00
No Intervention	22.82	46.76	3.53% (2.52%)	11.50% (3.32%)	$4,676.97	Dominated
Two-Stage Approach	23.04	47.4	0.00% (0.00%)	0.19% (0.09%)	$8,834.69	Dominated

© 2021 Wright JD et al. *JAMA Network Open.*

eFigure 6. 1-Way Sensitivity Analysis

U: utility, OC: ovarian cancer, RR: risk ratio, AC: all-cause, Hyst-BSO: hysterectomy with bilateral salpingo-oophorectomy. *Indicates no other strategy on efficiency frontier.

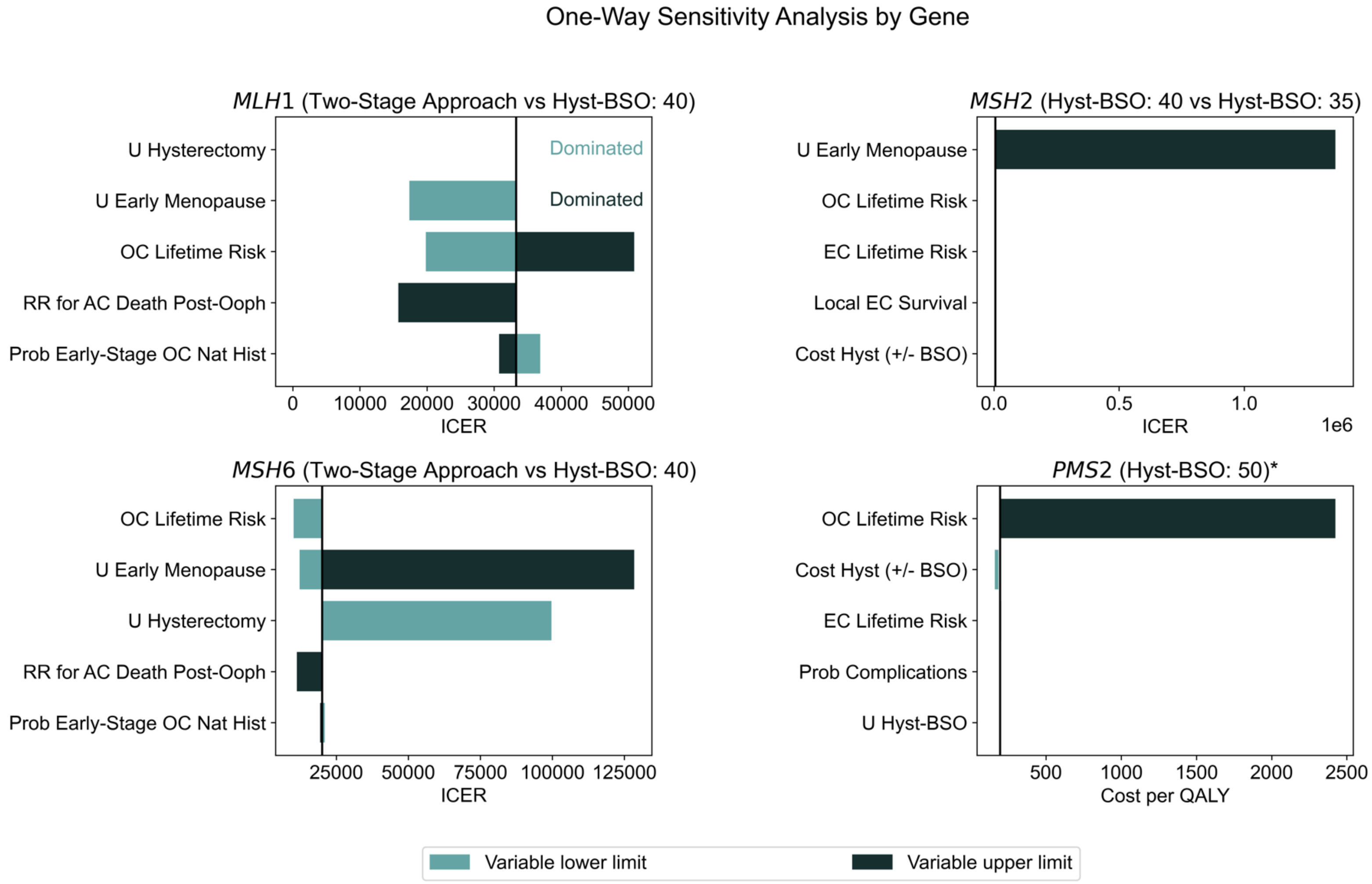

© 2021 Wright JD et al. *JAMA Network Open.*

eFigure 7. Threshold Analysis: Risk Ratio for Ovarian Cancer Development Postsalpingectomy.

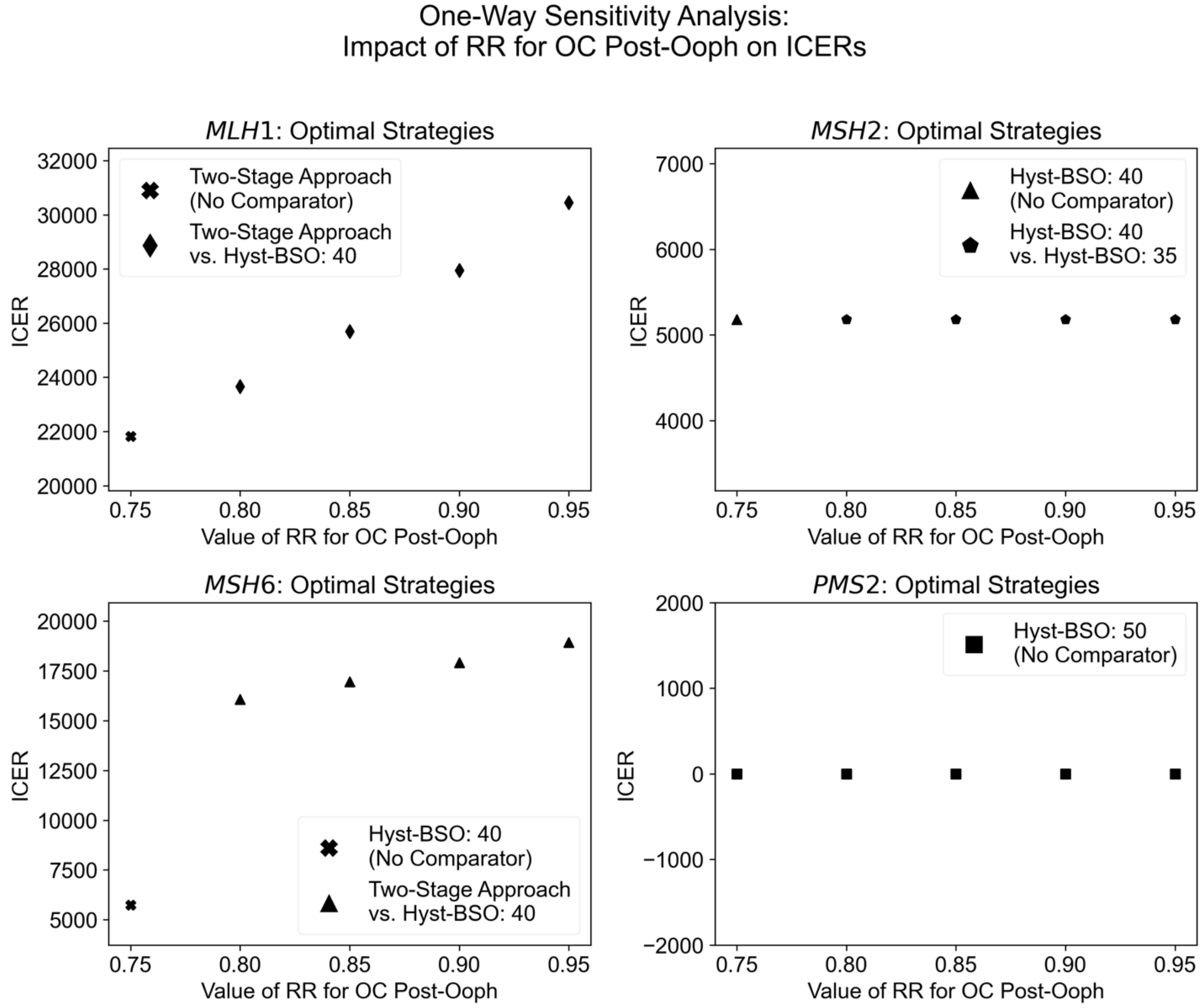

© 2021 Wright JD et al. *JAMA Network Open.*

eFigure 8. Threshold Analysis: Risk Ratio for All-Cause Mortality Postoophorectomy.

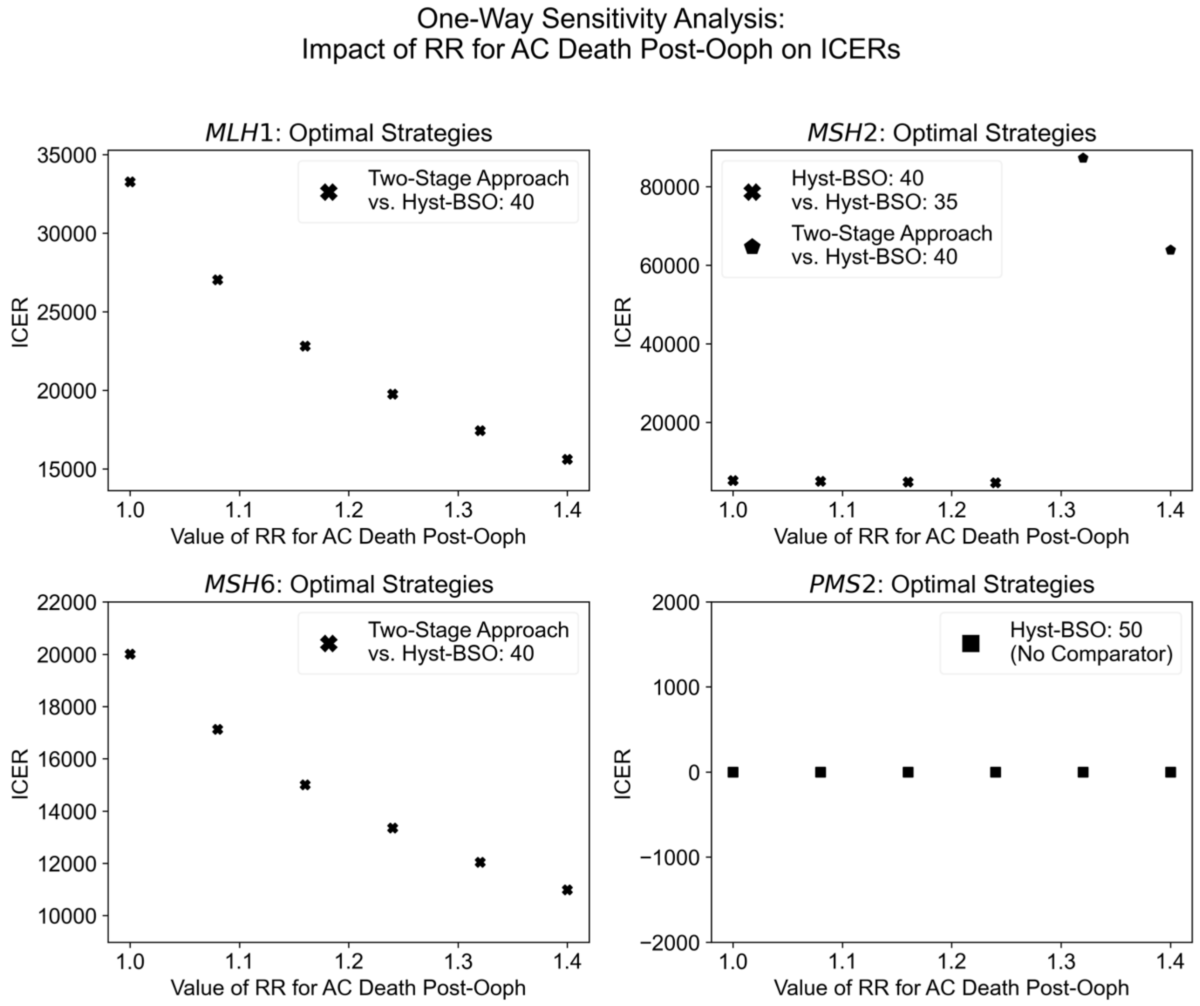

© 2021 Wright JD et al. *JAMA Network Open.*

eFigure 9. Threshold Analysis: Utility of Postoperation Status (Without Menopause)

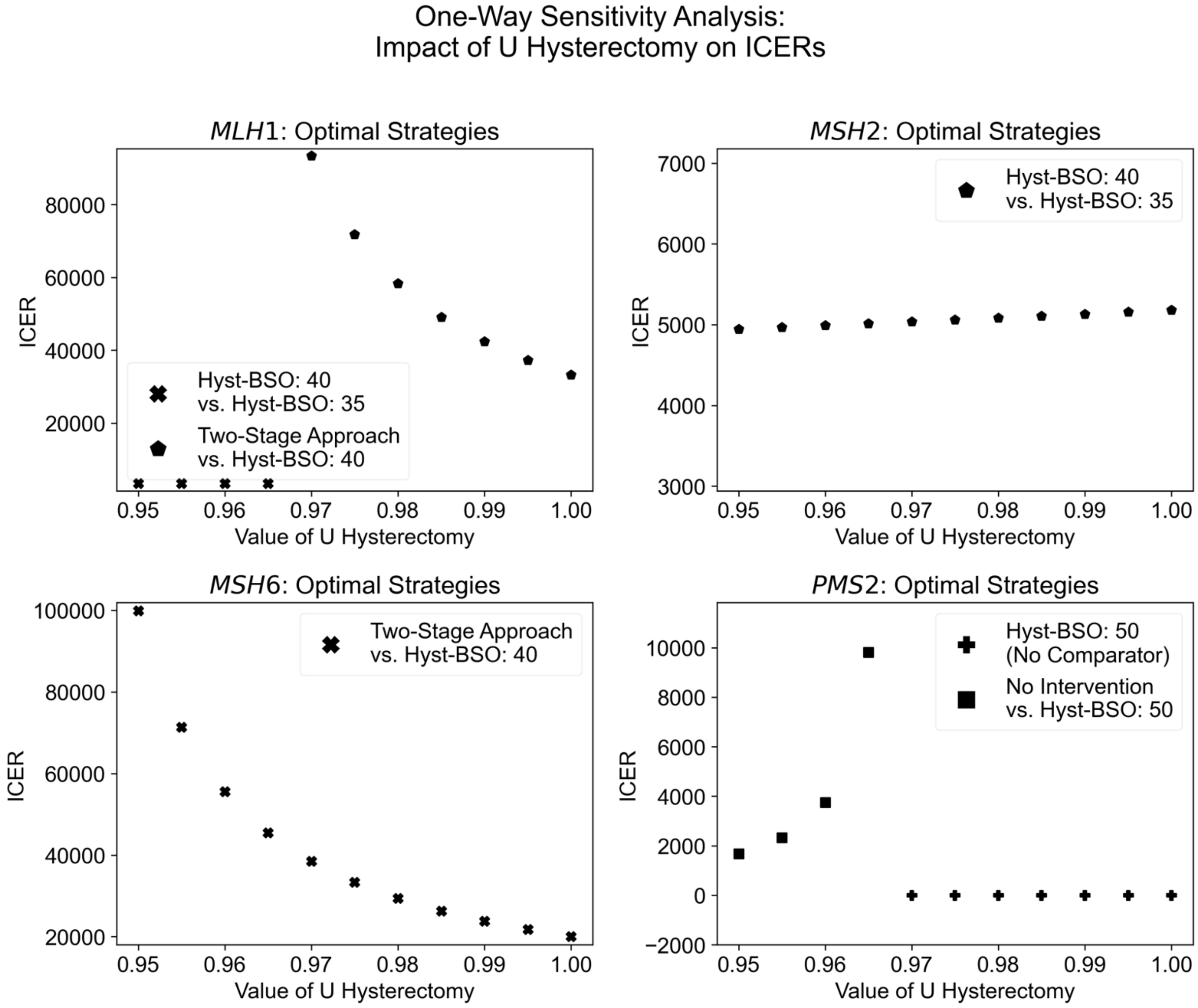

© 2021 Wright JD et al. *JAMA Network Open.*

eFigure 10. Threshold Analysis: Utility of Early Menopause

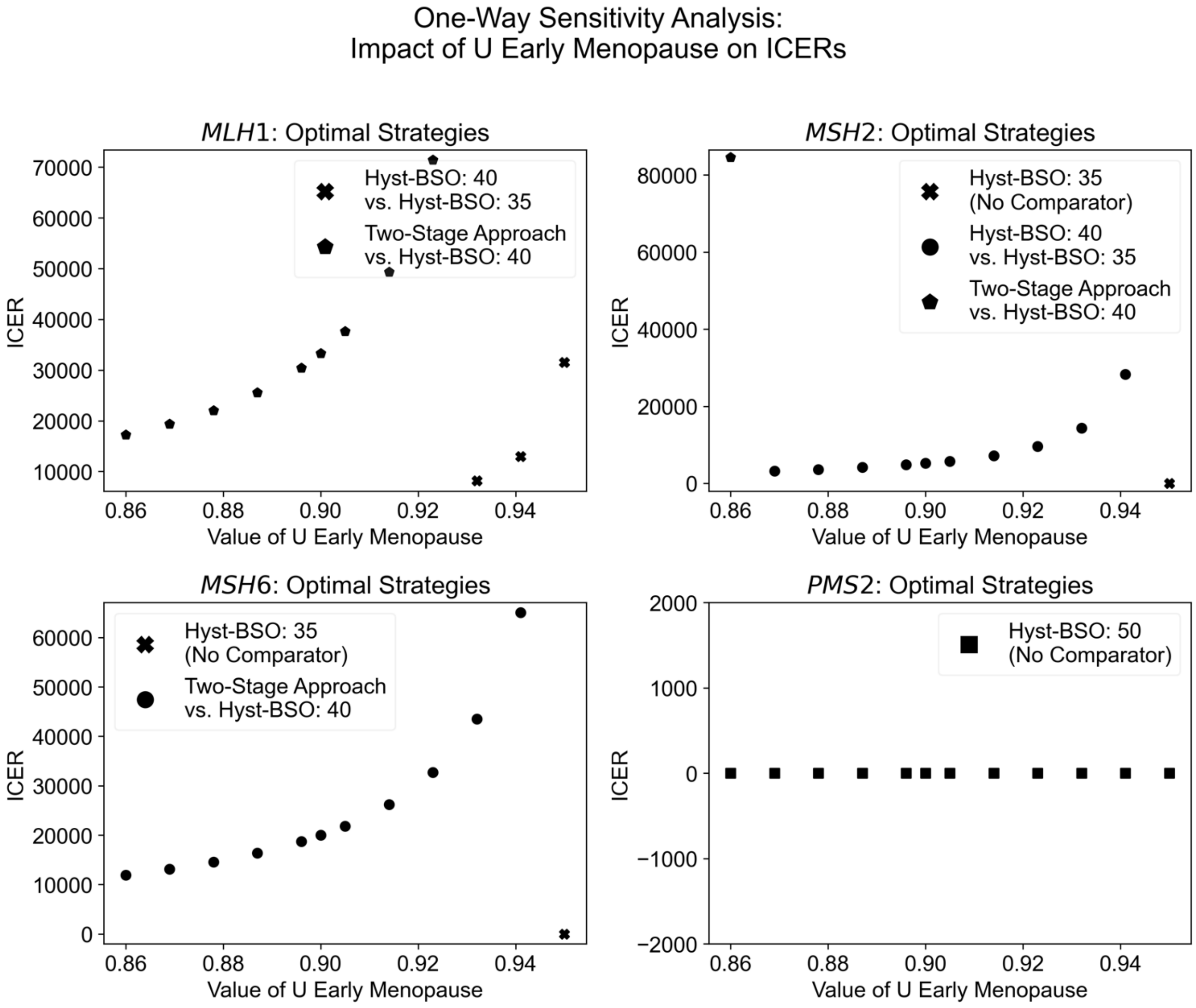

© 2021 Wright JD et al. *JAMA Network Open.*

eFigure 11. Cost-Effectiveness Acceptability Results for Probabilistic Sensitivity Analysis.
Hyst-BSO: Hysterectomy with bilateral salpingo-oophorectomy; Two-Stage Approach: hysterectomy and bilateral salpingectomy at age 40, bilateral oophorectomy at age 50; WTP: willingness-to-pay threshold.

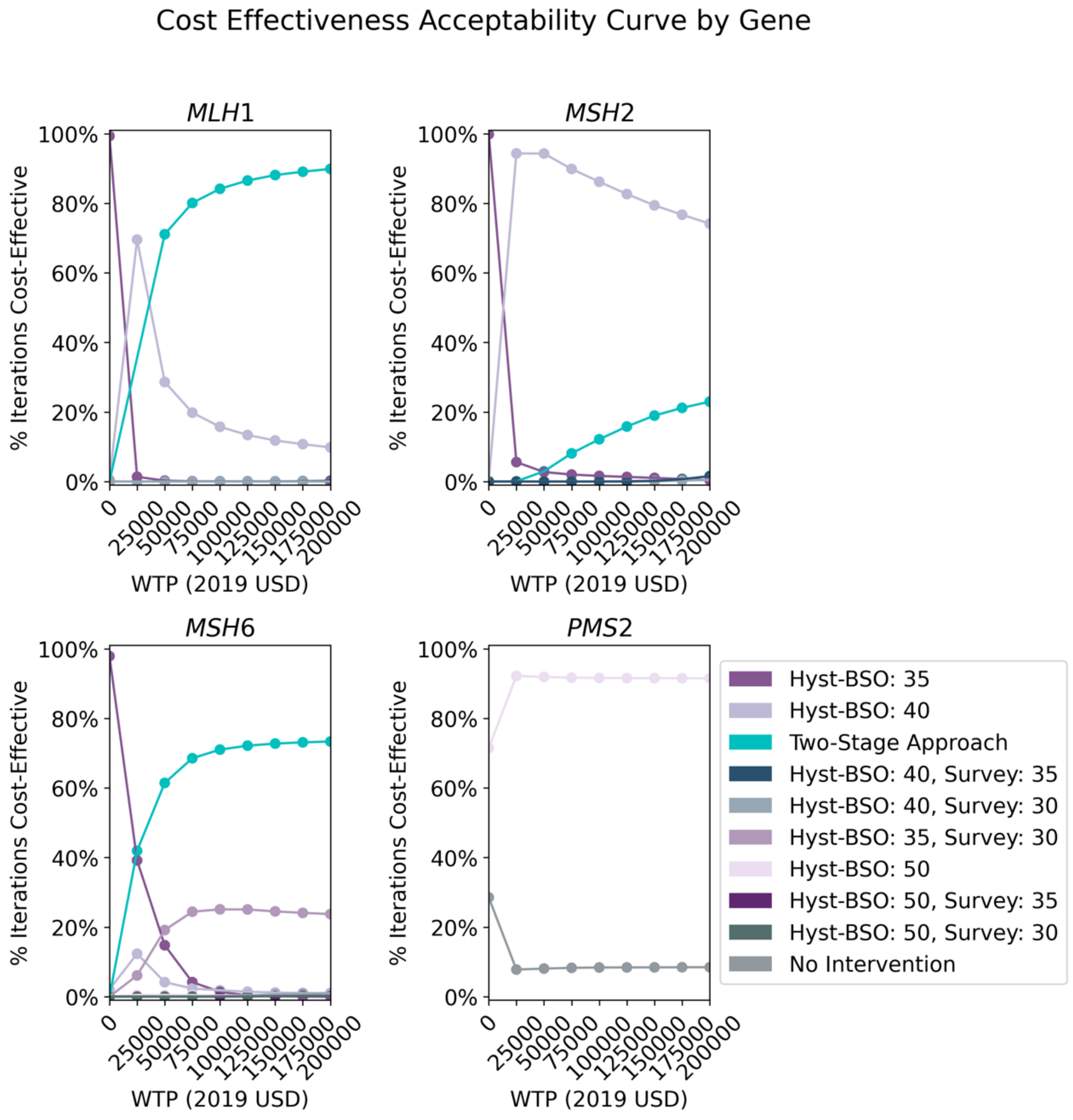

© 2021 Wright JD et al. *JAMA Network Open.*

eTable 5. Variation in Base Case Results From 10 000 Probabilistic Sensitivity Analysis Samples

	**Average QALYs (SD)**	**Average Life-Years (SD)**	**Average Total Cost (SD)**	**Average ICER (SD)**	**% of Trials on Efficiency Frontier**
MLH1					
Two-Stage Approach	22.35 (0.19)	45.96 (0.26)	$16,202.33 ($1,330.29)	$76,363.31 ($751,494.84)	90.65%
MSH2					
Hyst-BSO: 40	22.11 (0.17)	46.43 (0.25)	$10,547.38 ($1,256.55)	$8,046.15 ($12,314.92)	99.65%
MSH6					
Two-Stage Approach	21.97 (0.48)	45.14 (1.06)	$19,658.65 ($5,126.58)	$46,349.96 ($514,972.49)	96.28%
PMS2					
Hyst-BSO: 50	22.17 (1.0)	45.61 (2.12)	$13,859.86 ($11,400.27)	$1,977.76 ($19,590.17)	99.83%

© 2021 Wright JD et al. *JAMA Network Open.*

eReferences

1. Dominguez-Valentin M, Sampson JR, Seppälä TT, et al. Cancer risks by gene, age, and gender in 6350 carriers of pathogenic mismatch repair variants: findings from the Prospective Lynch Syndrome Database. *Genetics in Medicine*. Published online July 24, 2019:1-11. doi:10.1038/s41436-019-0596-9

2. ten Broeke SW, Brohet RM, Tops CM, et al. Lynch syndrome caused by germline PMS2 mutations: delineating the cancer risk. *J Clin Oncol*. 2015;33(4):319-325. doi:10.1200/JCO.2014.57.8088

3. Yang KY, Caughey AB, Little SE, Cheung MK, Chen L-M. A cost-effectiveness analysis of prophylactic surgery versus gynecologic surveillance for women from hereditary non-polyposis colorectal cancer (HNPCC) Families. *Familial Cancer*. 2011;10(3):535-543. doi:10.1007/s10689-011-9444-z

4. Havrilesky LJ, Moss HA, Chino J, Myers ER, Kauff ND. Mortality reduction and cost-effectiveness of performing hysterectomy at the time of risk-reducing salpingo-oophorectomy for prophylaxis against serous/serous-like uterine cancers in BRCA1 mutation carriers. *Gynecologic Oncology*. 2017;145(3):549-554. doi:10.1016/j.ygyno.2017.03.025

5. Yabroff KR, Lamont EB, Mariotto A, et al. Cost of Care for Elderly Cancer Patients in the United States. *J Natl Cancer Inst*. 2008;100(9):630-641. doi:10.1093/jnci/djn103

6. Bhattacharya S, Middleton LJ, Tsourapas A, et al. Hysterectomy, endometrial ablation and Mirena® for heavy menstrual bleeding: a systematic review of clinical effectiveness and cost-effectiveness analysis. *Health Technol Assess*. 2011;15(19):iii-xvi, 1-252. doi:10.3310/hta15190

7. Roberts TE, Tsourapas A, Middleton LJ, et al. Hysterectomy, endometrial ablation, and levonorgestrel releasing intrauterine system (Mirena) for treatment of heavy menstrual bleeding: cost effectiveness analysis. *BMJ*. 2011;342:d2202. doi:10.1136/bmj.d2202

© 2021 Wright JD et al. *JAMA Network Open.*
